# Functional changes in hippocampal synaptic signaling in offspring survivors of a mouse model of intrauterine inflammation

**DOI:** 10.1186/s12974-017-0951-1

**Published:** 2017-09-05

**Authors:** Melissa H. Kelley, Wendy W. Wu, Jun Lei, Michael McLane, Han Xie, Kyle D. Hart, Leonardo Pereira, Irina Burd, James Maylie

**Affiliations:** 1 Obstetrics and Gynecology, Oregon Health & Science University, Portland, OR 97239 USA; 2 Integrated Research Center for Fetal Medicine, Gynecology and Obstetrics, Johns Hopkins University, Baltimore, MD 21287 USA; 3 Present address: US Food and Drug Administration, Silver Spring, USA

**Keywords:** Intrauterine inflammation, Hippocampus, Synaptic transmission, Long-term synaptic potentiation

## Abstract

**Background:**

Recent evidence suggests that exposure to intrauterine inflammation causes acute fetal brain injury and is linked to a spectrum of neurobehavioral disorders. In a rodent model of intrauterine inflammation induced by lipopolysaccharide (LPS) exposure in utero, activated microglia can be detected in the hippocampus of offspring survivors, as late as 60 days postnatal (DPN). Given that the hippocampus is important for learning and memory, these results suggest that in utero inflammation underlies long-term cognitive deficits observed in children/survivors.

**Methods:**

An established mouse model of LPS-induced intrauterine inflammation was used to study hippocampal function from offspring at 44–59 DPN. Microgliosis was examined at 45 DPN. Extracellular field recordings of synaptic transmission were performed on acute hippocampal slices.

**Results:**

LPS offspring mice displayed persistent microglial activation and increased CA3–CA1 excitatory synaptic strength, which can be explained in part by an increase in the probability of glutamate release, and reduced long-term synaptic potentiation compared to control mice.

**Conclusions:**

These results offer a mechanistic explanation for the cognitive and behavioral deficits observed in survivors of preterm birth caused by intrauterine inflammation.

## Background

Pregnancy and gestation are a critical time for fetal development. Recent evidence suggests that fetal exposure to inflammatory cytokines can have long-lasting effects on postnatal physiology, sometimes lasting into adulthood [1, 2]. During intrauterine infection/inflammation, proinflammatory cytokines and other mediators can cross the compromised blood–brain barrier and induce activation of microglia and signaling through astrocytes in the fetal brain, causing subsequent production of reactive oxygen species and cytokines that can lead to glutamate-induced excitotoxicity [3–5]. In addition to the possibility of modulation of neurodevelopment, moderate maternal inflammation also leads to elevated production of serotonin in the placenta, which can disrupt fetal neurodevelopment of serotonin-dependent processes in the forebrain [6].

Preterm children that are exposed to inflammation in utero are at a greater risk for neurological, emotional, and learning disorders [7–9]. Additionally, maternal inflammation has been linked with increased prevalence of autism and experimental animal models mimicking maternal infection and inflammation result in autism-like phenotypes [10, 11]. Similarly, elevated levels of maternal cytokines, in particular tumor necrosis factor (TNF)α, were associated with increasing odds of adult schizophrenia and other psychoses in their offspring [12]. These results strongly suggest that in utero inflammation and postnatal cognitive abnormalities are causally linked.

Animal models of intrauterine inflammation include mice, rats, rabbits, and sheep [13, 14]. One well-characterized rodent model of preterm birth that mimics human situation of local inflammatory response in the uterus and no overt infection in the dam is intrauterine lipopolysaccharide (LPS) injection [13]. At high doses (250 μg/100 μl), LPS injection results in > 95% preterm birth and significant fetal brain injury [1, 15]. Decreasing LPS doses (50 μg/100 μl), however, resulted in ~ 30% preterm births, yet with detectable levels of activated microglia as late as 60 days postnatal (DPN) [2, 16]. In mice exposed to LPS in utero, the volume of the hippocampus, a structure important for learning and memory, is reduced. Whether hippocampal functions are altered in the survivors of preterm birth is currently unclear. In this study, we tested whether exposure to low-dose LPS in utero alters information transfer and storage by the hippocampus in adult survivor mice to understand the cellular mechanisms contributing to cognitive deficits in survivors of preterm birth.

## Methods

### Mouse model of intrauterine inflammation

All animal care and treatment procedures were approved by the Institutional Animal Care and Use Committee, and animals were handled according to the National Institutes of Health guidelines. An established model of intrauterine inflammation was utilized for these studies [15]. Briefly, timed pregnant CD-1 outbred mice were obtained from Charles River Laboratories (Wilmington, MA). Intrauterine injections of 100 μl of LPS (from *Escherichia coli*, 055: B5, Sigma-Aldrich, St. Louis, MO) at a dose of 50 μg in 100 μL of phosphate-buffered saline (PBS) were administered on embryonic day 17 (E17) of a 19-day gestation period in four independent experiments. Control dams for these experiments received the same volume of intrauterine injection of vehicle. In total, 11 dams were injected with PBS with all litters surviving and 43 dams were injected with LPS with 16 litters surviving. For survival surgery, pregnant mice were anesthetized using isoflurane, and a mini-laparotomy was then performed in the lower abdomen for intrauterine injections. Live pups were separated by sex, and only males were utilized for these studies. While sex could play an important role in the long-term effects of intrauterine inflammation [2], we concentrated on males only in the current study to avoid possible effects of estrus cycle in which circulating hormones could affect hippocampal function [17] as well as potential sex difference in microglia during development [18].

### Immunohistochemistry

At 45 DPN, after the animals were euthanized, 1× PBS was perfused transcardially, followed by 4% paraformaldehyde (PFA). The brain from one animal from each dam was dissected and post-fixed in PFA overnight. The next day, specimens were washed with PBS extensively and immersed in 30% sucrose until saturation, followed by cryosection at 20-μm thickness. Sections were incubated overnight at 4 °C with rabbit anti-Iba-1 (Wako, Richmond, VA) to identify microglia. Donkey anti-rabbit Dylight 568 (Abcam, Cambridge, MA) was applied as the secondary antibody. The sections were further counter-stained with DAPI (Roche, Indianapolis, IN) to identify cell nuclei. Images were obtained using an Axioplan 2 Imaging system (Carl Zeiss, Thornwood, NY) at the bregma level from − 1.34 to − 1.70 mm. Quantitative analysis of Iba-1 expression cell numbers and area percentage within CA3–CA1 was performed using ImageJ 1.37V (NIH). Each cell was identified as the positive expression (red) in cytoplasm and DAPI (blue) in nucleus. The percentage of Iba-1 expression area was calculated by positive expression area (cell bodies and branches) divided by CA3–CA1 area. The average number of both hippocampi in each hemisphere represented the section counted. The average number of five sections represented the specimen counted.

### Hippocampal slice preparation

Mice were anesthetized with isoflurane and rapidly decapitated. The brain was removed, and 300-μm slices from the middle of the hippocampus were cut using a vibrating microtome (VT1000S; Leica Instrument, Leitz) as the brain was immersed in an ice-cold sucrose substituted artificial CSF (aCSF) of the following composition (in mM): 119 NaCl, 26 NaHCO_3_, 2.5 KCl, 1 NaH_2_PO_4_, 1 MgCl_2_, 2 CaCl_2_, and 25 dextrose (oxygenated with a carbogen mixture of 95% O_2_ and 5% CO_2_). Slices were held in oxygenated aCSF at 35 °C for 30 min and then at room temperature (22–24 °C) for at least 1 h before recording.

### Electrophysiology

All recordings were made at room temperature. Hippocampal slices were visualized using a fixed-stage, upright microscope (Axio Examiner or Leica DMLFS) equipped with infrared differential interference contrast optics. The recording chamber was continuously perfused with oxygenated aCSF flowing at a rate of 1–2 ml/min. Recording electrodes were pulled from borosilicate pipettes (Sutter Instruments) and had tip resistances of 2–3.5 MΩ when filled with aCSF for extracellular field recordings. Glass stimulating electrodes of approximate resistance of 1 MΩ were filled with aCSF, connected to a Digitimer constant current stimulus isolation unit (AutoMate Scientific, Berkeley, CA), and placed in the middle of the CA1 stratum radiatum to stimulate the CA3 axon collaterals. The CA3 axons were severed to eliminate recurrent excitation within the CA3 subfield. The stimulating and recording electrodes were placed in the middle portion of the CA1 stratum radiatum (approximately equal distance from stratum pyramidal and stratum lacunosum moleculare). Stimulus duration was 0.1–0.2 ms allowing for clear separation of fiber volley (FV) from the preceding stimulus artifact. Long-term potentiation (LTP) of CA3–CA1 excitatory synapses was induced by stimulating CA3 axons with 3 sets of 100 stimuli delivered at 50 Hz.

Recordings were obtained using a Multiclamp 700B amplifier (Molecular Devices) or GeneClamp 500 amplifier (Axon Instruments). Signals were filtered at 3 kHz, digitized using a Digidata 1440A interface (Molecular Devices) at 10 kHz, and transferred to a computer using pClamp10 software (Molecular Devices) or an ITC-16 (Instrutech Corp., NY) and a computer using Patchmaster (Heka Instruments).

All experiments were performed in the presence of picrotoxin (100 μM) and CGP55845 (2 μM; both from Tocris Bioscience) to suppress inhibitory synaptic transmission.

#### Data and statistical analyses

Voltage traces were analyzed using custom macros written in Igor Pro (WaveMetrics). Statistical analysis was done using R version 3.3.2 and the geepack package [19]. To account for correlation among hippocampal slices taken from the same animal, we constructed simple GEE models of field excitatory postsynaptic potential (fEPSP) input–output (I/O) relationship, FV–stimulus intensity, paired pulse ratio, and long-term synaptic plasticity with exchangeable correlation structures and, to accommodate the small number of mice relative to the number of slices, a jackknife variance estimator, with intrauterine exposure to LPS as the explanatory variable. We compared Iba-1 expression cell numbers and area percentage using Student’s *t* test with unequal variance.

#### Availability of data and materials

All data generated or analyzed during this study are included in this published article. Custom macros written in Igor Pro (WaveMetrics) are available on request (JM).

## Results

### Immunohistochemical evaluation

**Fig. 1:**
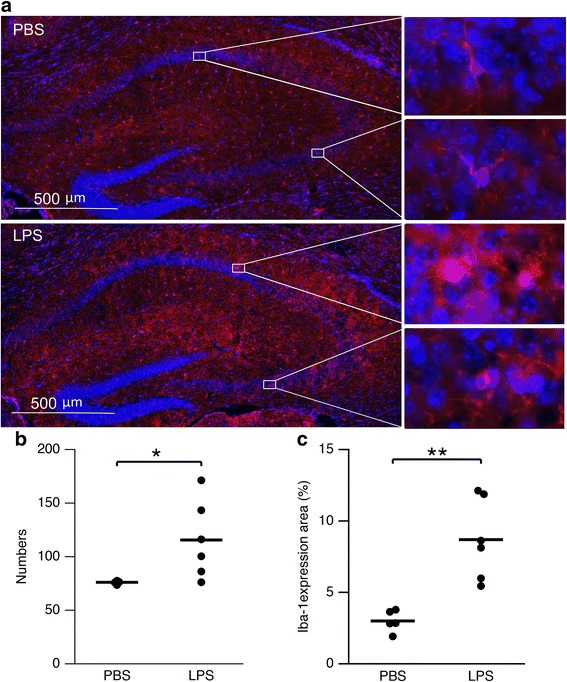
LPS exposure in utero generated long-term changes in glial appearance in the hippocampus on 45 DPN. **a** Micrograph showing increased microgliosis (red) in the hippocampus of LPS-exposed mouse. Insets on the right show enlarged image of CA1 and CA3 area. **b** Scatterplot of microglia cell number in the hippocampus from PBS (*n* = 5)- and LPS (*n* = 6)-exposed mice. Asterisks indicate *p* < 0.05. **c** Scatterplot of Iba-1 expression area in the hippocampus from PBS (*n* = 5)- and LPS (*n* = 6-exposed mice. Asterisks indicate *p* < 0.01

Mice were exposed to LPS in utero at E17 via intrauterine injection of 50 μg LPS in normal saline. Microgliosis was examined in brain cryosections on 45 DPN by immunohistochemistry using antibodies to Iba-1 (microglia) and DAPI (cell nuclei) (Fig. 1a). In CA3–CA1 of the hippocampus, Iba-1 expression cell number was significantly higher in intrauterine LPS-exposed pups compared to control (115.5 ± 36.1, *n* = 6 versus 76.1 ± 1.6, *n* = 5, *p* = 0.044, Fig. 1b). The percentage of Iba-1expression area was also significantly higher among intrauterine LPS-exposed pups (8.695 ± 2.831, *n* = 6 versus 3.002 ± 0.750 *n* = 5, *p* = 0.003, Fig. 1c). LPS-treated microglia demonstrated a round shape with fewer branches (amoeboid), indicating the activation of microglia.

### Exposure to LPS in utero causes an increase in excitatory synaptic strength in adult offspring

**Fig. 2:**
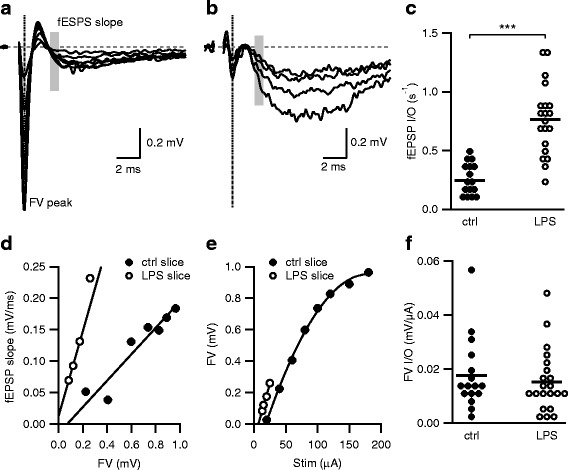
Exposure to LPS in utero causes a long-term increase in synaptic strength. **a**, **b** Representative fEPSPs evoked by increasing stimulation intensities from hippocampal slices obtained from adult control mice (**a**) and mice exposed to LPS in utero (**b**). Each trace is the average of five consecutively recorded voltage traces. Gray bars highlight the regions where fEPSP initial slopes were measured. Dashed line illustrates the point where the FV peak was measured. The stimulus artifact preceding the FV was blanked out. **d** fEPSP slope versus FV relation from a single representative experiment. fEPSP initial slope versus FV relations were fit with linear functions without constraints. The slope derived from the fits (s^−1^) reflects the input–output relation of synaptic transmission (fEPSP I/O). **c** Scatterplot of fEPSP I/O determined in **d** from individual experiments for control- and LPS-exposed mice. Asterisks indicate *p* < 0.001. **e** FV versus stimulus strength (Stim) relationship for a single representative experiment representing the FV input–output relationship (FV I/O). The FV I/O was fit with a quadratic function to calculate the slope of the FV I/O at threshold (FV I/O slope). **f** Scatterplot of FV I/O slope determined in **e** for individual experiments demonstrate that CA3 intrinsic excitability and axonal density were not significantly different

Acute brain slices were prepared from adult male survivors of LPS exposure and extracellular fEPSPs were measured for CA3–CA1 synapses. Increasing stimulus intensities resulted in increases in the fiber volley (FV) and fEPSP amplitudes. As FVs reflect activation of the CA3 axons (presynaptic effect), the initial slope of the fEPSPs reflects activation of the α-amino-3-hydroxy-5-methyl-4-isoxazolepropionic acid (AMPA) receptors (postsynaptic effect); the fEPSP slopes (shaded bar) were plotted against the FV amplitudes (dashed line) to yield a fEPSP input–output (I/O) relation (Fig. 2a). The fEPSP I/O relations constructed from individual experiments were fit with linear functions, and they reflect presynaptic action potential (AP) generation and the postsynaptic membrane response due to presynaptic AP-induced glutamate release (Fig. 2d). Therefore, we used the slope of the fEPSP I/O relationship as an indicator of synaptic strength (Fig. 2c). Mice that were exposed to LPS in utero displayed a robust increase in synaptic strength (estimated using a GEE model) compared to controls (LPS 0.76 (95% CI 0.58 to 0.94), *n* = 20, from 7 mice, 5 litters; control 0.24 (95% CI 0.15, 0.34), *n* = 15, from 5 mice, 5 litters; *p* < 0.001). These results demonstrate that intrauterine inflammation resulted in increased synaptic strength at hippocampal CA3–CA1 synapse.

These extracellular recordings also allowed for an examination of the CA3 presynaptic excitability as described by plotting the peak of the FV against the stimulus intensity (Fig. 1e). The FV–stimulus intensity relations showed no significant difference between LPS and control mice (Fig. 2f; LPS 0.016 (95% CI 0.008, 0.023), *n* = 20; control 0.018 (95% CI 0.016, 0.019), *n* = 15, *p* = 0.543). These results suggest that intrinsic excitability of the CA3 axon collaterals was not affected by intrauterine LPS exposure.

### Probability of glutamate release is affected in mice exposed to intrauterine LPS

**Fig. 3:**
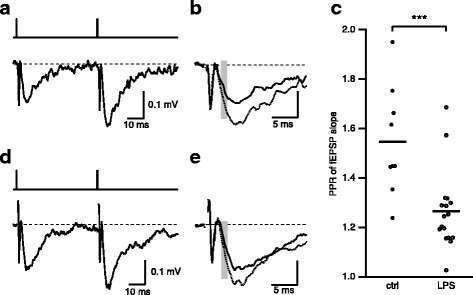
LPS exposure in utero causes reduced PPR. **a**, **b** Representative fEPSPs evoked by paired stimulation separated by 50 ms (top trace) from hippocampal slices obtained from adult control mice (**a**) and mice exposed to LPS in utero (**d**). Each trace is the average of 10 consecutively recorded voltage traces. **b**, **e** Overlay of the first (black) and second (dash line) fEPSPs. Gray bars highlight the regions where fEPSP initial slopes were measured. **c** Scatterplot of PPR determined by measuring the slope of the average of 10 individual trials. Asterisks indicate *p* < 0.001

The increase in fEPSP I/O observed could reflect either pre- and/or post-synaptic changes. To determine whether presynaptic effects on glutamate release contribute to the increased synaptic strength in LPS-exposed mice, extracellular paired-pulse measurements were made by giving two stimuli in close succession (50 ms) and measuring the slope of the average of 10 fEPSPs for the first and second responses (Fig. 3). The stimulus intensity used for these measurements was set to approximately 50% of the maximum evoked first response. LPS-exposed mice showed reduced levels of facilitation of the second pulse compared to control mice, as demonstrated by a reduction in the paired pulse ratio or PPR (LPS 1.27 (95% CI 1.19, 1.34), *n* = 17; control 1.54 (95% CI 1.44, 1.64), *n* = 9; *p* < 0.001; Fig. 3c). These results suggest greater release of glutamate induced by the first stimulus, hence a reduction in the amount of glutamate release by the second stimulus. Therefore, the increased synaptic strength observed in LPS-exposed mice is in part mediated by a change in the probability of glutamate release.

### Long-term synaptic potentiation is impaired in mice exposed to LPS in utero

**Fig. 4:**
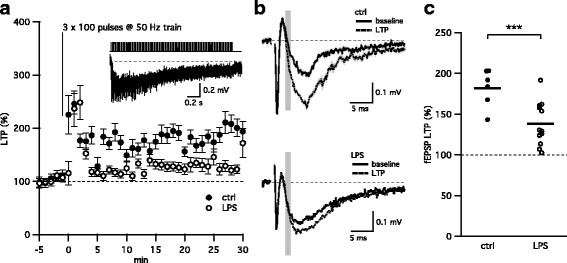
LPS exposure in utero reduces LTP formation. **a** Time course (mean ± SEM) of the normalized fEPSP response (slope) before (− 5–0′) and after LTP induction (0–30′). Gray line and inset above demonstrate a 50-Hz stimulation train and typical response elicited by LTP stimulation protocol (3 × 100 pulses at 50 Hz). **b** Representative average of 10 fEPSPs from a single experiment before (baseline, black) and after LTP induction (LTP, dash). Gray bars highlight the regions where fEPSP initial slopes were measured. **c** Scatterplot depicting the reduced levels of LTP observed in mice exposed to LPS in utero. Asterisks indicate *p* < 0.001

The hippocampus is required for memory formation, and long-term potentiation of the CA3–CA1 excitatory synapses is thought to be the cellular correlate that mediates hippocampus-dependent memory formation [20–22]. LTP at these synapses involves a rapid increase in synaptic strength that is largely attributed to increased post-synaptic AMPA receptor insertion [23–25]. The degree of LTP-induced at these synapses was compared between LPS-exposed and control mice. Extracellular fEPSPs were measured at CA3–CA1 synapses using the same protocol as for I/O function (Fig. 2), with the initial stimulus intensity set to give 25–50% of the maximal field response. After achieving 5 min of stable baseline responses, a high-frequency train of stimuli (3 sets of 100 pulses at 50 Hz; Fig. 4a) was administered to induce LTP. The degree of LTP was measured 25–30 min after delivery of the high-frequency train. LPS-exposed mice showed reduced levels of LTP compared to control mice (LPS 138.3 (95% CI 123.7, 152.9), *n* = 12; controls 182.0 (95% CI 165.6, 198.3), *n* = 6; *p* < 0.001; Fig. 4. These results suggest inappropriate information storage/coding following LPS exposure.

## Discussion

Children born preterm due to exposure to intrauterine infection or inflammation are at greater risk for developing acute fetal brain injury as well as adverse neurological outcomes including cognitive, motor, and behavioral disabilities such as autism [13, 26–28]. Using a murine model of inflammation and perinatal brain injury, we demonstrated that offspring of pregnant mice exposed to LPS displayed increased synaptic strength, due in part to an increase in the probability of glutamate release from the presynaptic CA3 axon terminals, as evidenced by the reduced PPR ratio. LPS-exposed mice also had lower levels of LTP compared to control mice. This finding could be due to disrupted AMPA receptor trafficking in CA1 pyramidal neurons—a similar cellular change observed in ischemia-induced impairment of LTP [29]. Synaptic dysfunctions and impaired synaptic plasticity at the hippocampal synapses are commonly observed in animal models of disease or toxic substance exposure that exhibit cognitive deficits, including fragile X syndrome [30], Alzheimer’s disease [31], accelerated aging [32], and perinatal/acute exposure to lead and polychlorinated biphenyls [33] or marijuana [34]. Thus, the novel findings from the present studies showing changes in hippocampal synaptic transmission and plasticity offer a plausible explanation for the cognitive and behavioral deficits observed in survivors of preterm birth.

The cellular and molecular mechanisms of inflammation-induced fetal brain injury are not fully understood but are likely to involve proinflammatory chemokine and cytokine signaling. During intrauterine inflammation, infectious pathogens likely activate toll-like receptors (TLR) on the surface of cells in the decidua and placental membranes, resulting in the production of proinflammatory cytokines that can cross the compromised blood–brain barrier into the fetal brain where they activate microglia, the primary defense mechanism in the brain [4, 5]. This process then initiates a cascade of events that leads to increase in proinflammatory cytokines IL-1, IL-6, and TNFα that remain elevated after birth [35–37]. Evidence from adult inflammation models shows that microglia are activated and produce the proinflammatory cytokine TNFα that signals through astrocytes to irreversibly alter synaptic transmission and impair cognition [38–40]. The precise mechanism by which TNFα causes increased synaptic transmission has been studied extensively and may include both pre- and post-synaptic effects that involve retrograde signaling of prostaglandins and nitric oxide [41–43] and synaptic scaling [40]. Importantly, activation of microglia and release of TNFα and the subsequent signaling through astrocytes is the key event leading to behavioral comorbidities as a result of chronic inflammation. Indeed, chronic administration of the microglial/macrophage activation inhibitor minocycline to the inflamed animal both lowered the level of TNFα in the hippocampus and completely abolished the effect of peripheral inflammation which induced changes in synaptic transmission and synaptic plasticity [44].

Interestingly, peripheral inflammation is a feature of many adult neurodegenerative diseases and is often associated with marked behavioral changes, including mood disorders, fatigue, cognitive and memory dysfunction, and sleep disturbances. Moreover, the inflammation is capable of aggravating other neurological and neuropsychiatric conditions, including seizure disorders, major depression, Alzheimer’s disease, multiple sclerosis, Parkinson’s disease, and autism [45]. The changes in synaptic transmission in adult inflammation models are similar to those observed here with prenatal inflammation. It is likely that the persistent activation of microglia 45 DPN in survivors of LPS-treated mice in utero contributes to these changes. Whether they are mediated by TNFα signaling is yet to be determined.

This study not only demonstrates the effects of inflammation on brain function but also reveals a unique long-lasting component of the effect of inflammation into adulthood when experienced during a critical period of development. In addition to having implications for neurological effects from peripheral inflammation, these findings extend our understanding of the cognitive deficits in those born preterm as well as other disorders such as autism and schizophrenia.

## Conclusion

Using a murine model of inflammation and perinatal brain injury, we demonstrated that offspring of pregnant mice exposed to LPS displayed altered hippocampal excitatory synaptic function. Synaptic transmission at CA3–CA1 synapses was increased due in part to an increase in the probability of glutamate release from the presynaptic CA3 axon terminals. Importantly, LPS-exposed mice also had lower levels of LTP compared to control mice. These novel findings offer a plausible explanation for the cognitive and behavioral deficits observed in survivors of preterm birth caused by intrauterine inflammation.

## Abbreviations

aCSF: Artificial cerebral spinal fluid
AMPA: **α**-Amino-3-hydroxy-5-methyl-4-isoxazolepropionic acid
AP: Action potential
DPN: Days postnatal
fEPSP: Field excitatory postsynaptic potential
FV: Fiber volley
I/O: Input–output
LPS: Lipopolysaccharide
LTP: Long-term potentiation
PPR: Paired pulse ratio
TLR: Toll-like receptors
TNF: Tumor necrosis factor
